# INCLUSION AND INTEGRATION OF COMMUNITY STAKEHOLDER PERSPECTIVES IN RESEARCH: INSIGHT FROM AND FOR GERONTOLOGISTS

**DOI:** 10.1093/geroni/igad104.1906

**Published:** 2023-12-21

**Authors:** Carrie Leach, Karen Moss

## Abstract

Engaging diverse stakeholders in research is increasingly required by funding agencies though training, skills and capacity building for engaged approaches have not kept pace. To advance the science of community engaged research (CEnR) we convene leading scholars who will provide insight on the inherent issues of engaging stakeholders in research and strategies for overcoming obstacles. The first presentation will summarize findings from a study of gerontologists and aging trainees to understand the needs and challenges of engaging patient and community stakeholders in research. The second will present strategies for engaging Certified Nursing Assistants (CNAs) in a communication program for people living with dementia in skilled nursing facility. The third will present findings from community discussions that informed adaptations to the development of remote sensing technologies and discuss the continuing role for community engagement in implementation. The fourth presentation will offer insight on integrating diverse perspectives in the development of a dementia-focused intervention in a nursing home. The final presentation will summarize the results of a tobacco exposure study to inform the development of a harm-reduction intervention among long-term renters in extended-stay hotels and the benefits of stakeholder partnerships in building trust and aiding in renter recruitment. We will conclude with an attendee inclusive discussion to envision a path that may empower people of all ages and at all career stages to engage diverse stakeholders as partners to catalyze scientific research that is responsive and relevant to those who have a stake in healthy aging and results in social change. This is a collaborative symposium between the Community-Engaged Research and Patient/Person Engagement in Research Interest Groups.

